# Skull fractures by glass bottles tested on cadaveric heads

**DOI:** 10.1007/s00414-023-03133-8

**Published:** 2023-12-19

**Authors:** Ana I. Lorente, Samuel Maza-Peón, César Hidalgo-García, Carlos López-de-Celis, Jacobo Rodríguez-Sanz, Albert Pérez-Bellmunt, Mario Maza-Frechín

**Affiliations:** 1 Instituto Universitario de Investigación en Ingeniería de Aragón (I3A), Universidad de Zaragoza, C/Mariano Esquillor s/n, 50018 Zaragoza, Spain; 2 Center for Applied Biomechanics, University of Virginia, 4040 Lewis and Clark Drive, Charlottesville, VA 22911 USA; 3 Researching Unit of Physiotherapy, University of Zaragoza, c/Domingo Miral s/n, 50009 Zaragoza, Spain; 4 Universitat Internacional de Catalunya, Actium Functional Anatomy Group, Faculty of Medicine and Health Sciences, C/Josep Trueta, s/n, 08195 Sant Cugat del Valles (Barcelona), Spain

**Keywords:** Head injury, skull fracture, blunt force, glass bottles, human tolerance, biomechanics

## Abstract

Head trauma is frequently related to the misuse of drinking vessels as weapons. Forensic reports usually evaluate these blunt injuries as having occurred in scenarios where the alcohol intake is high. Fatal consequences are seen in blows with glass bottles aiming at the head. To prove the outcome that a glass bottle thrown to the head could cause, three intact human cadaver heads were impacted with 1-liter glass bottles at 9.5 m/s using a drop-tower. The impact location covered the left temporal bone, sphenoid bone, and zygomatic arch. The contact between the head and the bottle was produced at an angle of 90° with (1) the valve of the bottle, (2) the bottom of the bottle, and (3) with the head rotated 20° in the frontal plane touching again with the bottom of the bottle. The three bottles remained intact after the impact, and the injury outcomes were determined by computed tomography (CT). The alterations were highly dependent on the impact orientation. The outcome varied from no injury to severe bone fractures. In the most injurious case (#3), fractures were identified in the cranial base, sphenoid bone, and zygomatic bone. These testing conditions were selected to replicate one specific legal case, as required by the plaintiff. Physical disputes with bar glassware can lead to complex combinations of blunt and sharp-force injuries. Controlled biomechanical studies can benefit forensic analyses of violence involving glassware by providing a better understanding of the underlying injury mechanisms.

## Introduction

Bottles and glasses are used as weapons in physical disputes [1]. The injury outcome of blows with bar glassware often consists of lacerations and incise wounds [2–4]. However, the misuse of bar glassware also leads to skull fractures that can be associated with life-threatening scenarios [5]. Half-liter beer bottles have been described as formidable weapons in human skull fractures due to their breaking energy thresholds [6]. Forensic practical casework can benefit from biomechanical knowledge and research about skull injuries caused by blows with glassware [2, 5].

This study was required by a court to replicate a real case where the victim avoided being hit by a bottle that was thrown at his head. The victim’s reflexes avoided a potential injurious scenario. The thrown bottle broke the car window behind the victim’s head. This is not the first research study required for a legal proceeding with regards to bottles and head injuries: Bolliger et al. (2009) studied whether full and empty bottles can cause skull fractures at the request of a court [6].

Impact testing with post-mortem human surrogates (PMHS) allows us to simulate the use of forces found in regular traumatic events to understand the human response to external loads [7, 8]. PMHS have been used to define injury criteria and to understand skull fractures since the 1940s [9]. Loads can be applied by a wide range of testing setups to replicate the impact scenarios, such as drop-towers performing free falls [6, 7, 10].

Impact tests on PMHS skulls have shown complex fracture patterns with wider fracture widths away from the loading site [8]. This effect of dissipating energy through the cranium at a different point away from where the impact occurred has been described as “secondary fractures”, as opposed to the “primary fracture” that occurs at the impact point [11]. In physical disputes, skull fractures are related to the energy required to break bar glassware. Once the stein breaks, severe sharp trauma is also a risk [5].

Prevention initiatives to reduce bar glassware injuries have been proposed in the literature. Replacing glass with plastic would substantially decrease injuries and their cost [1]. Another suggestion has been to increase the toughness of bar glassware: replacing pint glasses with toughened glassware has been linked to lower injury risk in a randomized controlled trial [12, 13]. The toughness of glassware found in bars varies widely, with impact resistances ranging from 0.08 J to more than 4 J [14]. A better knowledge of the biomechanics related to glassware injuries would help to understand the mechanical forces causing these injuries and, therefore, better decisions about how to reduce these injuries could be made.

The goal of this study was to represent the potential head injuries that a 1-liter glass bottle could have caused in a real prosecuted dispute. The hypothesis was that the event could have caused severe skull fractures if the bottle had impacted the victim’s head.

## Materials and methods

Three cryopreserved human heads (3 males, Table 1) were tested and CT-scanned before and after testing. The heads were disarticulated at the C6–C7 cervical level and stored at −25°C. They were thawed at room temperature (22°C) 24 h prior to testing. All specimens were free of intra-articular and bone pathologies. Approval was obtained from the ethics committee Comitè d’Ètica de Recerca-Universitat Internacional de Catalunya (Ref. CBAS-2023-07). The protocol followed was in line with the Declaration of Helsinki.

**Table 1:** Demographic data

ID	Sex	Age (year)	Cause of death
#1	Male	69	Cardiopulmonary arrest
#2	Male	63	Cardiopulmonary arrest
#3	Male	78	Oncologic

**Fig. 1:**
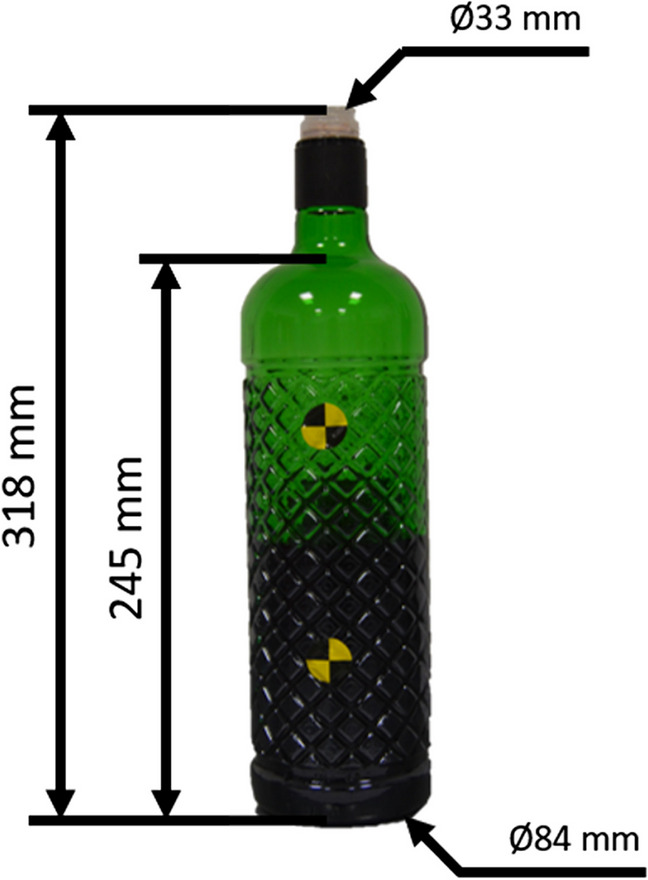
The bottles were half-full in all the tests

Three 1-liter glass bottles of sloe brandy were used, testing each of them in one different head specimen. The bottles were half-full (Fig. 1) with a weight of 1.1 Kg (Table 2). The height of the bottles was 318 mm, and 245 mm until the bottle-neck; the base diameter was 84 mm, and the plastic on the mouth of the bottle had a diameter of 33 mm (Fig. 1). This bottle was selected to replicate the specific legal case.

**Table 2:**
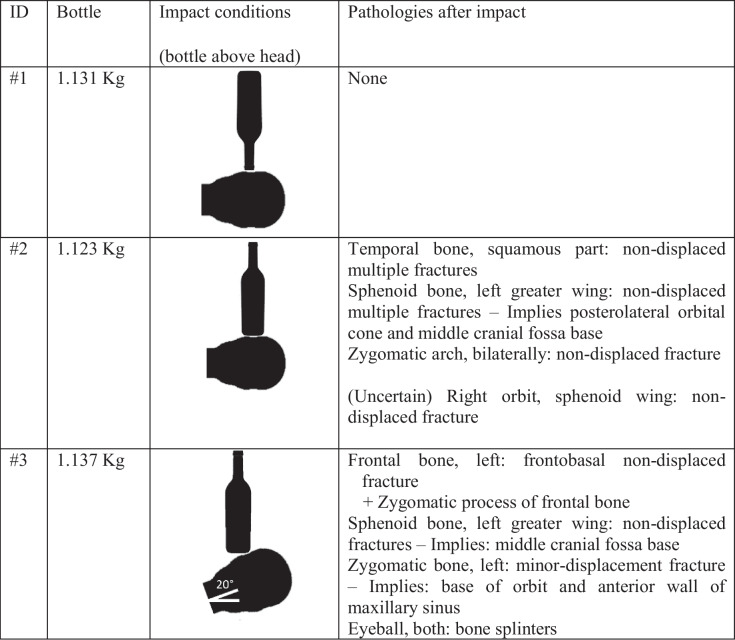
Test conditions and post-test pathologies seen in CT

**Fig. 2:**
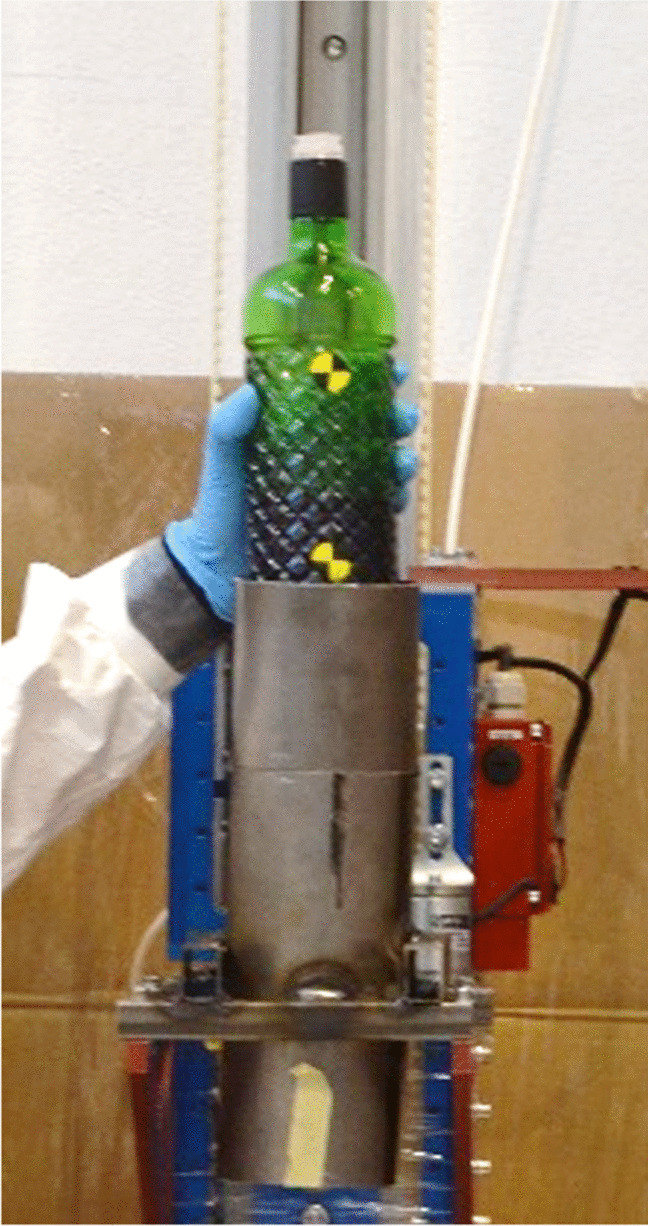
Metallic cylinder where the bottle was placed for the free-fall impacts

The impact on the heads was caused by a free-fall guided impact machine (Quebrantahuesos 6.0, +D, Spain). The bottle descended with a metallic cylinder supported inside it by paper tape (Fig. 2). The three bottles had an impact speed of 9.5 m/s (error: −0, +0.15 m/s). This was the estimated speed of the bottle thrown in the conditions of the real event: a man of 1.7 m, handling the bottle at his neck height and being 1 m away from the victim’s head [15]. This event resulted in a kinetic energy of 49.6 J. The heads under the impact machine were positioned to have the impact on the left lateral area (temporal bone, sphenoid bone, and zygomatic arch). The heads were positioned on a soft foam rubber to allow free inertial reaction [16] with the following orientations: the head was horizontal and the bottle impacted vertically with the mouth of the bottle, which had a plastic valve (case #1), the head was again horizontal and the bottle impacted vertically with its circular base (case #2), and, in the last scenario, case #2 was modified by rotating the head 20° in the frontal plane, having the neck lower than the top of the head and 70° between the head and the bottle (case #3), as illustrated in Table 2.

## Results

**Fig. 3:**
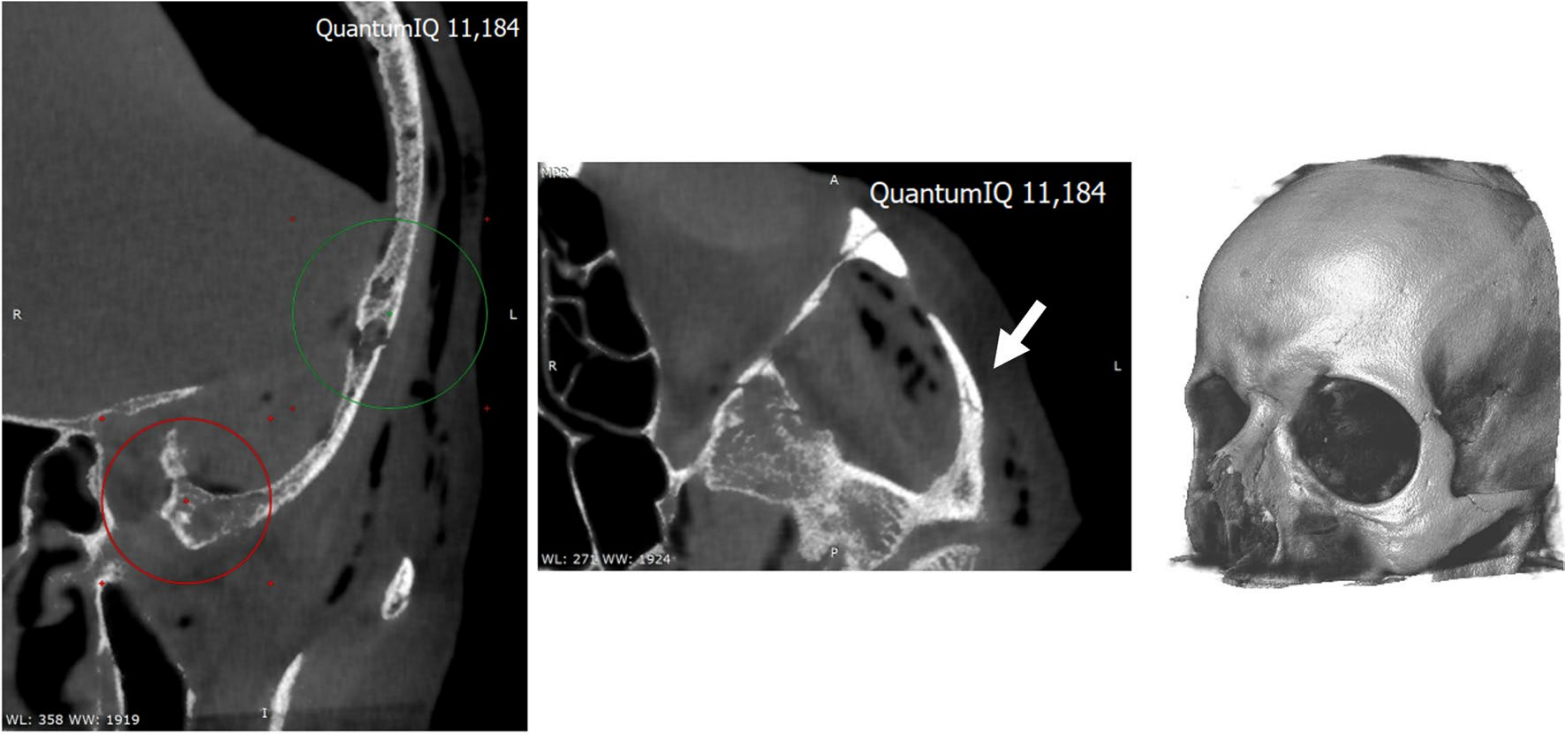
Case #2 showed fractures on the left temporal bone (left image) and left zygomatic arch (right image)

**Fig. 4:**
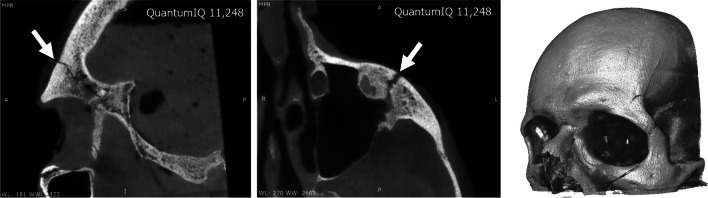
Case #3 showed left frontobasal fractures (left image) and a fracture on the left zygomatic bone (right image)

The impact conditions were different for each head, which led to different injuries (Table 2). In the first case (#1), the contact was with the plastic valve instead of with glass. This first case showed no injury on its CT images, although the mark from the impact was visible on the skin. Case #2 showed injuries on the temporal bone, sphenoid bone, and zygomatic arch (Table 2, Fig. 3). An arch mark with the shape of the bottle’s base was visible on the skin. Case #3 had a lower contact area than case #2 due to the head’s rotation with respect to the bottle (20°, Table 2). The conditions of case #3 led to fractures on the frontal bone, the sphenoid bone, and the zygomatic bone (Table 2, Fig. 4). These injuries would have had severe physical consequences.

## Discussion

The impact tests with 1-liter glass bottles presented in this study caused severe injuries in one out of the three cases. The test conditions replicated a real case of a court, by request of the plaintiff. The authors were only able to find another similar study in the literature which was also requested for a court [6]. This other study by Bolliger et al. (2009) used a drop-tower setup as well, but with 0.5-liter glass beer bottles. Although only one mechanical study related to a request by a court was found, many forensic cases show head injuries caused by drinking vessels [1–3, 5]. It is possible to find other mechanical studies related to head injuries caused by drinking vessels but without being related to legal procedures [14, 17, 18] or mechanical studies focused on the properties of the skull [8, 10, 19, 20].

When mechanical testing aims to widen the knowledge about skull fractures caused by drinking vessels, useful conclusions for the forensic field can be reached. Some studies support that drinking vessels cannot cause head injuries; Nentwig et al. (2021) revealed that cranial fractures in adults are unlikely with 0.5-liter glass beer bottles or 0.33-liter empty glass Coke bottles due to their maximum energy transmission [18]. This conclusion was reached by hitting bottles against a dummy head covered with an acrylic scalp surrogate or pork rind. This outcome is in line with the findings of Madea et al. (2004), who performed postmortem blow tests on the vertex area with three different types of bottles (0.7–0.8 L) and found no injuries in any of their 20 cases [2]. But it should be noted that in their 20 cases, all the bottles broke with the impact. Furthermore, in the forensic cases reported by Madea et al. (2004), mechanical damage to the skull was rare and the fatal events were only related to lacerations of the scalp [2]. Their mechanical study also concluded that the filling conditions of the bottles did not influence their testing outcome [2]; but this disagrees with the study by Bolliger et al. (2009) that found different breaking energies for empty (40 J) and full bottles (30 J) [6]. These energies of 30 J and 40 J are higher than the energy levels that cause injuries on the temporal, parietal, and zygomatic bones (14.07 J) [8]. Fractures can result from glass bottle blows, as evidenced in the present study in cases #2 and #3, proving that the risk of skull fractures exists in blows to the head with glass bottles.

Although some studies have concluded that glass bottles would break before producing damage to the skull, injuries caused by drinking vessels have been reported in the literature: throwing a beer stein can cause skull fractures in the parietal and petrous bones [5, 17]. Furthermore, it cannot be ignored that nonlife-threatening skull fractures can lead to fatal complications, such as hemorrhages and inflammatory reactions [5]. When analyzing skull fractures due to drinking vessels, not only should the area of the impact be of interest, as injuries have been shown in areas remote from the impacted area [8]. This never happens with lacerations, as they are always on the striker area [20]. Therefore, bony injuries can be remote from where lacerations are seen. Soft tissue injuries should be expected [5] and brain injuries can also occur without bony fractures [16], as it has been seen with 1-liter stein traumas [17].

Cases #1 and #2 have a very low probability of occurrence in real situations as the bottles almost always move with a hammer-like motion, which reduces the likelihood of impacts having 90° between the head and the bottle, as described in these two cases (Table 2). To see the conditions of these two cases, the bottle should be thrown at the head’s height and in those specific orientations with respect to the head, and no rotation should occur in the bottle’s trajectory. Furthermore, the bottle we used to replicate the specific legal case had a plastic valve, but other glass bottles of alcoholic beverages are without any plastic valve and the contact in a scenario like case #1 would be with glass instead of with plastic. Case #2 showed a more severe scenario as the impact area (55.4 cm^2^) was larger than in case #1. Case #3 was the most harmful and could lead to permanent injuries.

Testing conditions should always be as close as possible to real assaults. The impacted area was selected to replicate a real case, which involved one of the most common regions compromised in assaults with glass steins: the temporo-parietal region [17]. The outcome varies widely depending on the affected anatomical location [8]. Furthermore, in these physical disputes, the velocity of the stein always depends on the ability of the assailant and his motivation [17]. But similar speed values can be observed in different studies: our estimated value of 9.5 m/s is close to the 12.5 m/s measured with two male volunteers without any special training [17]. In other biomechanical analyses that are not related to vessels blunt traumas the impact speeds ranged from 5.3 to 8.0 m/s [8, 11, 21]. Another similarity in testing conditions when comparing previous studies is the design of the head support. Soft foam rubber supports have been used to sustain the head while allowing free inertial reactions during impact testing [16]. On the other hand, it has been reported that different restraint levels in blunt force impacts have not influenced fracture forces, describing negligible differences in the energy thresholds between fixed and free configurations [10]. Testing on dry bones would influence the fracture patterns, as they would be irregular and random as opposed to perimortem-fresh specimens that would show clearly defined patterns [11]. The scalp conditions and skull thickness must be also considered, as they influence the injury patterns [2]. Lastly, the sex of the specimens should be carefully stated in biomechanical studies as female skull bones have shown lower strength values than male bones in postmortem impact testing [16]; and in most of the assaults, the victims are males [1, 17].

Testing conditions do not always consider the energy [22], and there is a lack of research relating the level of trauma to the amount of energy involved [20]. Sometimes, only the force is stated [18] or the force with other parameters such as speed [17] or acceleration [23]. Energy values can be found in some studies [6, 8, 10, 11, 19–21]. The importance of reporting energy values can be seen if we compare our testing conditions (9.5 m/s and 49.6 J) with other studies. Ribeiro et al. (2020) used a lower speed (5.44 m/s) but more than double the energy (118.4 J) [11]. From the information reported by Bolliger et al. (2009), the speed can be calculated (8.2 m/s), and although it was close to our speed, the energy value was much lower (30 J), and in their second scenario, the speed was larger (14.3 m/s), but the energy (40 J) was still lower than our value [6]. Lastly, the same can be observed in the study by Mole et al. (2015), a similar speed (9.34 m/s) had much lower energy (8.72 J) than our value of 49.6 J; and to reach higher kinetic energy (54.06 J) their speed increased to 23.25 m/s [20].

The breaking energies of vessels are related to the years they have been in use: used vessels have lower breaking energies [17]. Furthermore, complex glassware–head interactions are seen with the misuse of glassware as weapons, with the combination of blunt and sharp force trauma [5]. The present study was only able to consider bony injuries, but skin lacerations are the most common injury with 1-liter beer steins [17] and can lead to fatal outcomes due to exsanguination [2, 24]. These complex scenarios cannot be fully studied with PMHS and could have occurred in the real judicial scenario. Moreover, it is not possible to predict brain injuries using the present experimental setup either.

Brain injuries should be considered in these scenarios of blunt trauma, although severe brain injuries caused with glass bottles are rarely observed [2]. No evidence of blunt brain injuries were found in 1288 injurious cases which involved bottles or glasses [1]. In the present work, brain injuries were not studied as they cannot be replicated with cryopreserved PMHS. The brain boundary conditions in these PMHS differ from the conditions of living subjects [19]. The lack of information about the brain response is a common limitation when analyzing skull fractures with experimental setups [6]. Furthermore, brain injuries cannot be predicted from skull fractures [25]. Computational models facilitate the study of brain injuries [26], although some computational studies focused on skull biomechanics disregard the brain due to its complexity as a highly deformable element [27].

Other limitations may be the advanced age of the three heads tested (63, 69, and 78 years), and the limited number of tests performed (three). Aging changes have been observed in female skulls, but our three donors were male and cortical thinning remained the same in male skulls ranged from 20 to 99 years [28]. However, morphological changes have been measured in male skulls with aging [29], which could influence its response to traumatic events. Lastly, our number of impact tests was very limited with only three skulls representing three different scenarios. A much larger number of impact conditions and human skulls (trying different anatomical areas) should be performed due to the lack of knowledge about the potential injuries in violent assaults with drinking vessels. The area of impact determines the injury outcomes, as other areas, such as the frontal bone, require higher breaking energies [7, 8]. The number of experiments with human cadaveric heads and drinking vessels is very limited [2].

As in our case, experimental results from postmortem studies can be highly valuable in specific trial courts. The present study can be of interest in the analysis of glasses injuries in the head; although, compared to bottle injuries, glass injuries less often involve the scalp [1]. Furthermore, it must also be acknowledged that these kinds of experimental testing provide valuable data for the development of computational models [23, 30]. Computational models of finite element methods are a promising tool to evaluate different scenarios of head impacts in forensic routines [23, 26]. Forensic reports and computational biomechanics would benefit from more biomechanical studies focused on different impact scenarios with bar glassware.

## Conclusion

Skull fractures could be caused by 1-liter glass bottles thrown aiming at the head. Two out of our three impact tests, which covered the left temporal bone, sphenoid bone, and zygomatic arch, produced skull fractures, resulting in severe physical consequences in one case (case #3). The literature lacks experimental tests with cadaveric heads and bar glassware and an understanding of whether skull fractures can be caused by blows with glass bottles. Therefore, the experimental setup presented herein can also serve as an inspiration of how real-life scenarios can be replicated in biomechanics laboratories to better understand potential injurious cases and improve computational models. Injury biomechanics can provide valuable knowledge for forensic analyses, as our study did for a specific legal case.

## Data Availability

The post-test CT files are available on https://osf.io/6rgbj/?view_only=a96ee6d4ce6343e48c6885768a70c096.
